# Routinization of prenatal screening with the non-invasive prenatal test: pregnant women’s perspectives

**DOI:** 10.1038/s41431-021-00940-8

**Published:** 2021-08-13

**Authors:** Karuna R. M. van der Meij, Annabel Njio, Linda Martin, Janneke T. Gitsels-van der Wal, Mireille N. Bekker, Elsbeth H. van Vliet-Lachotzki, A. Jeanine E. M. van der Ven, Adriana Kater-Kuipers, Danielle R. M. Timmermans, Erik A. Sistermans, Robert-Jan H. Galjaard, Lidewij Henneman, the Dutch NIPT Consortium

**Affiliations:** 1 Department of Clinical Genetics and Amsterdam Reproduction and Development Research Institute, Amsterdam UMC, Vrije Universiteit Amsterdam, Amsterdam, The Netherlands; 2 Department of Midwifery Science, AVAG, Amsterdam Public Health Research Institute, Amsterdam UMC, Vrije Universiteit Amsterdam, Amsterdam, The Netherlands; 3 Department of Obstetrics and Gynaecology, Utrecht University Medical Center, Utrecht, The Netherlands; 4 VSOP—Patient Alliance for Rare and Genetic Diseases, Soest, The Netherlands; 5 Velp Midwifery Practice, Velp, The Netherlands; 6 Department of Public and Occupational Health, Amsterdam UMC, Vrije Universiteit Amsterdam, Amsterdam, The Netherlands; 7 Department of Clinical Genetics, Erasmus University Medical Center, Rotterdam, The Netherlands

**Keywords:** Genetic counselling, Human behaviour

## Abstract

Due to the favorable test characteristics of the non-invasive prenatal test (NIPT) in the screening of fetal aneuploidy, there has been a strong and growing demand for implementation. In the Netherlands, NIPT is offered within a governmentally supported screening program as a first-tier screening test for all pregnant women (TRIDENT-2 study). However, concerns have been raised that the test’s favorable characteristics might lead to uncritical use, also referred to as routinization. This study addresses women’s perspectives on prenatal screening with NIPT by evaluating three aspects related to routinization: informed choice, freedom to choose and (personal and societal) perspectives on Down syndrome. Nationwide, a questionnaire was completed by 751 pregnant women after receiving counseling for prenatal screening. Of the respondents, the majority (75.5%) made an informed choice for prenatal screening as measured by the multidimensional measure of informed choice (MMIC). Education level and religious affiliation were significant predictors of informed choice. The main reason to accept screening was “seeking reassurance” (25.5%), and the main reason to decline was “every child is welcome” (30.6%). The majority of respondents (87.7%) did not perceive societal pressure to test. Differences between test-acceptors and test-decliners in personal and societal perspectives on Down syndrome were found. Our study revealed high rates of informed decision-making and perceived freedom to choose regarding fetal aneuploidy screening, suggesting that there is little reason for concern about routinization of NIPT based on the perspectives of Dutch pregnant women. Our findings highlight the importance of responsible implementation of NIPT within a national screening program.

## Introduction

**Fig. 1:**
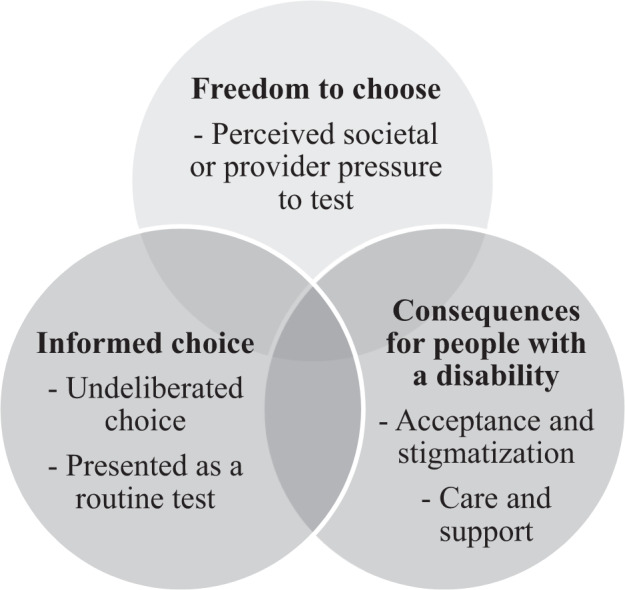
Three clusters of possible routinization of NIPT, adapted from Kater-Kuipers et al. [15]. The clusters comprise: Informed choice, Freedom to choose and Consequences for people with a disability.

Fetal aneuploidy screening allows couples to assess their risk for fetal anomalies (e.g., Down syndrome) and to make informed reproductive decisions (e.g., preparing for the birth of an affected child or terminating the pregnancy) [1]. In 2011, a new test was introduced for the detection of fetal aneuploidies: the non-invasive prenatal test (NIPT) [2]. NIPT is based on the analysis of cell-free DNA derived from maternal blood and has several key advantages compared to other screening methods such as the first-trimester combined test (FCT). NIPT has a higher sensitivity and gives fewer false-positives, thereby greatly reducing the need for confirmatory invasive tests that carry a risk of miscarriage and can be done early in pregnancy [3]. A high-risk NIPT result should nevertheless be confirmed with invasive diagnostic testing. The favorable characteristics of NIPT (accuracy, non-invasiveness, and early application) have led to a strong demand for implementation. Driven by both pregnant women and commercial industry [4], NIPT has disseminated quickly across the globe [5, 6]. Although studies have indicated positive attitudes toward NIPT from both pregnant women [7, 8] and healthcare professionals [8, 9], the introduction of NIPT has also raised profound ethical debates among patient organizations, professionals, and the public [10, 11]. There are concerns that the favorable characteristics of NIPT combined with its simple application may lead to the test becoming a routine part of prenatal care: offered to and accepted by pregnant women without proper counseling and/or consideration [12]. These concerns are often referred to as the “routinization” of fetal aneuploidy screening. Foster et al. [13] described routinization of genetic information as “a shift from being regarded as unique and exceptional, to being regarded as an ordinary aspect of routine medical research and care”. Already, NIPT has frequently been described by pregnant women as “just another blood test”, emphasizing the potential risk for routinization [14]. Kater-Kuipers et al. [15] distinguished various interpretations of the routinization concept used in scientific literature into three inter-related clusters: (1) informed choice, (2) freedom to choose, and (3) consequences for people with a disability (Fig. 1). First, informed choice has been referred to as women making the choice that is “based on relevant knowledge, consistent with the decision‐maker’s values and behaviorally implemented” [16]. It has been argued that the choice to accept or decline prenatal screening for fetal aneuploidy should be informed, because of the risks and ethics that are involved in the decision (e.g., invasive follow-up testing and the possibility of pregnancy termination) [17]. Questionnaire studies in the United Kingdom [18] and the Netherlands [19] have shown that the majority of high-risk women offered NIPT as a second-tier test following aneuploidy screening made an informed choice (89% and 77.9% respectively). However, in contrast to the reported high levels of informed choice, many women demonstrated misunderstandings regarding aspects of NIPT such as accuracy, conditions tested for [14], and test failure [20]. The ease of testing may challenge the decision-making process for pregnant women. To date, little is known about whether women who are offered NIPT as first-tier screening test make an informed choice and the factors that predict an informed choice. Second, freedom to choose involves arguments stating that the introduction of NIPT will lead to an increased uptake of fetal aneuploidy screening as participation becomes the norm, generating pressure on women to accept aneuploidy screening (Fig. 1) [15]. A survey study showed that most European healthcare providers anticipate a significant increase in NIPT uptake, primarily driven by women’s requests [9]. Professionals fear that the easy accessibility of NIPT might lead it to become self-evident and more difficult to decline [21]. A myriad of factors influence test uptake which may impede women’s freedom to choose, including the framing of the offer of screening, costs, and reimbursement policies [22]. However, it is still unclear how the introduction of NIPT as a first-tier test will influence women’s reasons to accept or decline screening, and whether women experience societal or provider pressure to test. Third, the consequences for people with a disability in the context of routinization signifies concerns that (more) screening with NIPT might lead to a decrease in the number of people with a disability, less available care and support, and an increase in discrimination and stigmatization, which may result in fewer women feeling free to decline prenatal screening (Fig. 1) [15, 23]. Despite the ethical debates regarding the routinization of NIPT, there is limited scientific evidence to support the concerns. With the application of NIPT continuing to become more widespread, it is important to consider women’s experiences and perspectives to ensure responsible implementation. In the Netherlands, the TRIal by Dutch laboratories for the Evaluation of Non-invasive prenatal Testing (TRIDENT-2) study examines the implementation of NIPT as a first-tier screening test for all pregnant women as part of a nationwide prenatal screening program for Down, Edwards, and Patau syndrome. Within TRIDENT-2, all pregnant women are offered a choice between NIPT, FCT, or no screening. An out-of-pocket payment is required for both tests; NIPT and FCT are offered at comparable costs of €175 and €168 (in 2018), respectively. All women are offered a pretest counseling session by a certified obstetric counselor. This survey study assessed whether there is evidence for concerns regarding the routinization of NIPT screening by examining women’s levels of informed choice, perceived freedom to choose, and personal and societal perspectives on Down syndrome.

## Materials and methods

This survey study is part of the TRIDENT-2 study and was approved by the VU University Medical Center Amsterdam Ethical committee (VUMC No. 2017.165).

### Study procedure

For this study, 28 midwifery practices and five hospitals assisted in recruiting respondents, who were distributed equally across the Netherlands to ensure a representative sample. Counselors handed out questionnaires to all pregnant women who received counseling for prenatal screening (NIPT and FCT) between September 2017 and October 2018. Counselors were offered a 25 euro gift voucher for their participation. Women were asked for consent to participate by their prenatal counselor, regardless of their screening choice (NIPT, FCT, or no test), and were given a package containing an information letter, two questionnaires, return envelopes and a pen.

### Questionnaires

A pre- and posttest questionnaire was developed by a multidisciplinary group of researchers including a representative from a patient organization, an obstetrician, a midwife, a clinical geneticist, and health scientists. The draft questionnaires were piloted using a think-aloud pretest [24] with five women and pretested among 44 pregnant women to explore feasibility and validity. The questionnaires were adjusted based on the feedback received. The first questionnaire was completed directly after pretest counseling. The second questionnaire was completed after receiving the results from the prenatal test (NIPT or FCT). The questionnaires were only available in Dutch. Here, we will describe results from the first questionnaire.

### Measures

The three clusters of routinization were operationalized using measures described below:

1. Informed choice: was assessed using the adapted multidimensional measure of informed choice [16, 25], combining the dimensions of knowledge, attitude, test uptake, and deliberation. Knowledge was measured using seven statements (answer options: “true”, “false”, or “do not know”), assessing knowledge regarding prenatal screening, NIPT, FCT, invasive testing, and the meaning of possible test results (Table S1) [26]. A cut-off of ≥5/7 correct questions was chosen to signify good knowledge (Table S2) [25]. Attitude was measured by asking respondents to score five bipolar adjective pairs regarding prenatal screening (bad–good; unimportant–important; frightening–not frightening; not reassuring–reassuring; not desirable–desirable) [27]. Sum scores were redistributed into three categories: positive (25–19), neutral (18–12), and negative (11–5). Respondents with a neutral attitude were excluded from the analysis of value-consistency as recommended in literature [18, 28]. Test-uptake was measured based on women’s intention to test, as the questionnaire was completed before the actual test. Respondents who indicated they were unsure of their choice were excluded from the analysis of informed choice (Table 2). Value-consistency was calculated by combining attitude and test uptake (intention) of NIPT or FCT. Deliberation was assessed using a six-item five-point Likert scale ranging from 1 (“strongly disagree”) to 5 (“strongly agree”) [28]. The mid-point (18) was used as the cut-off to dichotomize into a deliberated or not deliberated choice [25]. An informed choice was made if the decision was made with adequate knowledge, deliberated and behaviorally consistent with attitude (Tables 2 and S2).
2. Perceived freedom to choose included reasons for accepting or declining prenatal screening, measured by asking respondents to choose their most important reasons from a predetermined list (Table 3). Reasons for choosing NIPT or FCT was only completed by women who intended to opt for screening. The perceived societal pressure to test was measured on a two-item five-point Likert scale (Table 4). Respondents were asked whether they experienced societal pressure to accept and to decline prenatal screening.
3. Personal and societal perspectives on Down syndrome were assessed by presenting respondents with two statements: “Down syndrome is a serious condition” and “I would experience it as a heavy burden to raise a child with Down syndrome” (personal perspectives) and three statements: “are children with Down syndrome accepted in society”, “care and support for children with Down syndrome are well arranged”, and “parents are judged for having a child with Down syndrome” (perceived societal perspectives). Respondents could indicate their agreement on a five-point Likert scale ranging from 1 (“strongly disagree”) to 5 (“strongly agree”). Moreover, intention to terminate the pregnancy was measured on a two-item five-point Likert scale by asking women’s likelihood of choosing to terminate pregnancy in case of Down syndrome, or in case of Edwards or Patau syndrome.

Sociodemographic variables included were: maternal age, education level, ethnicity, religious affiliation, gestational age, parity and method of conception. Health literacy was measured based on a three-item set of brief screening questions [29, 30].

### Data analysis

All statistical analysis were done using IBM SPSS Statistics 22.0. Differences between groups were analyzed with chi-square tests for categorical variables and *t*-tests for continuous variables. Two logistic regression models were made to determine which variables predicted informed choice: a model including all variables (crude model) and a model using the backwards elimination method (adjusted model). A *p* value < 0.05 was considered significant.

## Results

In total, 752/1561 (48%) women agreed to participate in the survey study and returned the pretest questionnaire. Response rates varied strongly between participating midwifery practices/hospitals (2–89%, median 52%). The main reasons for non-response were a lack of interest, the questionnaire length, and language barriers. One questionnaire was excluded due to missing data. Characteristics of the 751 women are presented in Table 1. Mean age of the respondents was 31.6 years (SD 4.2), with a mean gestational age of 11.0 weeks (SD 2.0). The majority of respondents were highly educated (64.7%) and most were of Dutch descent (84.7%). The majority of the respondents intended to have fetal aneuploidy screening: 78.2% preferred NIPT and 2.0% FCT, 17.2% of women did not want a test, and 2.7% were unsure.

**Table 1:** Respondents’ characteristics.

	*n* (%)
Maternal age (missing 4)	
≤30	290 (38.8)
31–35	317 (42.4)
≥36	140 (18.7)
Education level (missing 4)^a^	
Low	38 (5.1)
Intermediate	226 (30.3)
High	483 (64.7)
Ethnicity (missing 4)^b^	
Dutch	633 (84.7)
Other western	61 (8.2)
Non-western	53 (7.1)
Religious affiliation (missing 10)^c^	
Not religious	496 (66.9)
Religious	245 (33.1)
Health literacy (missing 9)^d^	
Adequate	643 (86.7)
Not adequate	99 (13.3)
Gestational age (missing 5)	
≤10	285 (38.2)
11–14	425 (57.0)
≥15	36 (4.8)
Parity (missing 4)	
Nulliparous	372 (49.7)
Multiparous	376 (50.3)
Method of conception (missing 6)^e^	
Natural	671 (90.9)
Assisted	74 (9.9)
Screening intention	
NIPT	587 (78.2)
FCT	15 (2.0)
No test	129 (17.2)
Not sure	20 (2.7)

*FCT* first-trimester combined test, *NIPT* non-invasive prenatal test.

^a^Education levels categorized as low: elementary school, low level secondary school, or lower vocational training; intermediate: high-level secondary school or intermediate vocational training; high: high vocational training or university.

^b^Ethnicity categorized as Dutch: both parents were born in the Netherlands; other Western: one or both parents were born in Europe (excluding Turkey), North America, Oceania, Indonesia or Japan; non-Western: one or both parents were born in Africa, Latin-America, Asia (excluding Indonesia or Japan) or Turkey. Maternal country of birth was leading if both parents were born abroad.

^c^Religious affiliation was measured by the question “which denomination or ideology do you consider yourself as?” Answers were dichotomized: having no religious affiliation if answered “none” or having a religious affiliation if an affiliation was selected.

^d^Health literacy classified as inadequate if answered anything other than “never” or “occasionally” on one or more questions.

^e^Method of conception considered assisted: intrauterine insemination (*n* = 28), ovulation-induction (*n* = 21), in vitro fertilization (*n* = 11), intra-cytoplasmic sperm injection (*n* = 10) or preimplantation genetic diagnosis (*n* = 6).

### Informed choice

Tables 2 and S2 present a summary and description of the outcomes of the dimensions of informed choice. Overall, 83.2% of women (*n* = 619) had good knowledge about prenatal screening, 87.7% (*n* = 646) had deliberated their choice, and 99.2% (*n* = 522) made a choice behaviorally in line with their values. The majority of respondents (67.0%) had a positive attitude toward fetal aneuploidy screening. Overall, 75.3% made an informed choice about prenatal screening. The informed choice rate was higher for test-acceptors (76.8%) compared to test-decliners (59.6%). Of the 24.7% (*n* = 127) of women who made an uninformed choice, most women had either insufficient knowledge (47.2%, *n* = 60) or did not deliberate their choice (40.9%, *n* = 52) (Table 2).

**Table 2:** Dimensions of informed choice.

Knowledge	Deliberation	Attitude	Uptake^a^	*n* (%)
Informed choice				
Good	Deliberated	Positive	NIPT	351 (68.3)
Good	Deliberated	Negative	No test	28 (5.5)
Good	Deliberated	Positive	FCT	8 (1.5)
Uninformed choice				
Good	Not deliberated	Positive	NIPT	48 (9.3)
Insufficient	Deliberated	Positive	NIPT	46 (8.9)
Insufficient	Deliberated	Negative	No test	12 (2.3)
Insufficient	Not deliberated	Positive	NIPT	9 (1.7)
Good	Not deliberated	Positive	FCT	3 (0.6)
Insufficient	Deliberated	Positive	FCT	2 (0.4)
Insufficient	Not deliberated	Negative	No test	2 (0.4)
Good	Deliberated	Positive	No test	2 (0.4)
Insufficient	Deliberated	Positive	No test	1 (0.2)
Insufficient	Not deliberated	Positive	No test	1 (0.2)
Good	Not deliberated	Negative	No test	1 (0.2)

*FCT* first-trimester combined test, *NIPT* non-invasive prenatal test.

^a^Measured as intention to test.

Multiple logistic regression analysis showed that the variables religious affiliation and education level were significant predictors of informed choice, when correcting for maternal age, ethnicity, health literacy, parity, gestational age, and method of conception (Table S3). Respondents with an intermediate (OR: 3.37, 95% CI: 1.16–9.77 *p* = 0.025) or a high-level of education (OR: 3.29, 95% CI: 1.19–9.12, *p* = 0.022) were more likely to make an informed choice compared to women with a low level of education. Respondents with a religious affiliation were less likely to make an informed choice (OR: 0.58, 95% CI: 0.38–0.90, *p* = 0.015) compared to respondents without a religious affiliation.

### Perceived freedom to choose

The most important reasons for (intending) to accept (*n* = 1491) or decline (*n* = 271) prenatal screening are shown in Table 3. The main reasons for accepting prenatal screening were wanting reassurance that their child does not have Down, Edwards, or Patau syndrome (25.5%), and wanting as much information as possible about the health of their child (22.8%). Only 4.4% of respondents chose prenatal screening because their partner, family, or others wanted it, and 0.2% accepted prenatal screening because their obstetric healthcare professional thought it was a good idea. The most often reported reasons to decline prenatal screening were: every child is welcome (30.6%) and not wanting to terminate the pregnancy (21.0%). None declined because their obstetric healthcare provider conveyed it was not a good idea to participate.

**Table 3:** Reasons for accepting (*n* = 1491) or declining (*n* = 271) fetal aneuploidy screening.

Reasons for accepting screening	Responses (% of cases)	Reasons for declining screening	Responses (% of cases)
I want to be reassured that my child does not have Down, Edwards, or Patau syndrome	380 (25.5%)	Every child is welcome; a child with Down, Edwards, or Patau syndrome as well	83 (30.6%)
I want to have as much information as possible about the health of my baby	340 (22.8%)	I would never terminate my pregnancy	57 (21.0%)
I do not want to have a child with Edwards or Patau syndrome	257 (17.2%)	I think I have a low risk of having a child with Down syndrome	28 (10.3%)
I do not want to have a child with Down syndrome	186 (12.5%)	I am afraid I will regret testing when faced with an abortion decision	24 (8.9%)
I want to be able to prepare myself for the birth of a child with Down, Edwards, or Patau syndrome	145 (9.7%)	I think the tests are too expensive	17 (6.3%)
I am worried I will regret not testing later on	80 (5.4%)	I do not want to know if my child has a disorder	16 (5.9%)
My partner, family, or others want to test	66 (4.4%)	Because of my religion or faith	16 (5.9%)
Other	19 (1.3%)	I am not worried about my child’s health	14 (5.2%)
I think I have a high risk of having a child with Down syndrome	15 (1.0%)	I think the tests are not reliable^a^	7 (2.6%)
My midwife or doctor thinks it is a good idea	3 (0.2%)	I do not want to unnecessarily worry^a^	6 (2.2%)
		Other	3 (1.1%)
		My partner, family or others do not want to test	0 (0.0%)
		My midwife or doctor thinks it is not a good idea	0 (0.0%)

^a^Added reason based on other responses.

The most important reasons to choose either NIPT (*n* = 1652) or FCT (*n* = 29) among the 602 women intending to have screening are shown in Table S4. The main reasons for choosing NIPT were: NIPT is more reliable than FCT (29.1%), it is a safe test without a miscarriage risk (18.6%), and it is easy to do (16.0%). The main reasons to choose FCT were: the possibility to detect additional findings with ultrasound (31.0%) and because of the additional ultrasound for the nuchal translucency measurement (24.1%).

Of the *n* = 602 test-acceptors, 87.7% agreed that they did not feel pressured by society to accept prenatal screening. Similarly, 77.8% of the *n* = 126 women who declined screening agreed that they did not feel pressured by society to decline prenatal screening. Of test-acceptors, 7.4% reported pressure to decline, whereas 15.0% of test-decliners reported feeling societal pressure to accept screening (Table 4).

**Table 4:** Perceived societal pressure to test among test-acceptors (*n* = 602) and test-decliners (*n* = 129).

	(Totally) agree *n* (%)	Neither agree nor disagree *n* (%)	(Totally) disagree *n* (%)
Test-acceptors			
I feel societal pressure to accept screening	19 (3.2)	55 (9.2)	525 (87.6)
I feel societal pressure to decline screening	44 (7.4)	79 (13.2)	474 (79.4)
Test-decliners			
I feel societal pressure to accept screening	19 (15.1)	21 (16.7)	86 (68.3)
I feel societal pressure to decline screening	5 (4.0)	23 (18.3)	98 (77.8)

### Personal and societal perspectives on Down syndrome

**Fig. 2:**
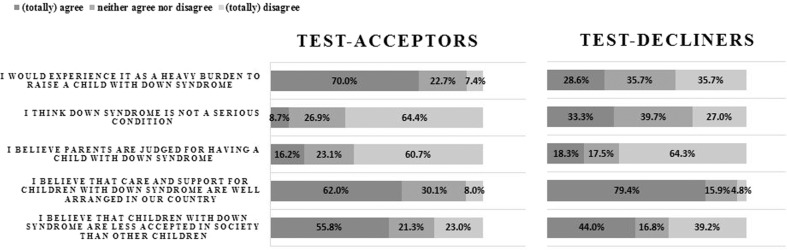
Personal and societal perspectives on Down syndrome of test-acceptors (*n* = 602) and test-decliners (*n* = 129). Level of agreement among test-acceptors and test-decliners regarding five statements.

Figure 2 shows that 70.0% of the test-acceptors indicated that they thought it would be a great burden to raise a child with Down syndrome, whereas only 28.6% of test-decliners agreed with this (*p* < 0.001). For both test-acceptors and test-decliners, the majority (60.7 and 64.3%) disagreed with the statement that parents of children with Down syndrome will be judged for having a child with Down syndrome (*p* = 0.692). Fewer test-acceptors (62.0%) than test-decliners (79.4%) agreed that care and support for children with Down syndrome are well arranged in the Netherlands (*p* < 0.001). Finally, more test-acceptors (55.8%) than test-decliners (44.0%) agreed that children with Down syndrome are less accepted in society than other children (*p* < 0.001). Among test-acceptors, the intention to terminate their pregnancy was lower for Down syndrome (50.1%) than for Edwards and Patau syndrome (79.2%).

## Discussion

This study describes women’s perspectives on different aspects of routinization after the introduction of first-tier NIPT within the TRIDENT-2 study. High levels of informed choice were found in our sample. The large majority of respondents did not perceive pressure to accept or decline screening. Significant differences between test-acceptors and test-decliners in personal and societal perspectives on Down syndrome were found.

### Informed choice

In this survey study, 75.3% of women offered NIPT made an informed choice for fetal aneuploidy screening. This number is comparable with results from a previous Dutch study among women offered FCT before the implementation of NIPT (75.5%) [26], and with results from the TRIDENT-1 study which was aimed at high-risk women choosing between NIPT and invasive testing (77.9%) [19]. In the UK, levels of informed choice were compared between a study setting (89.0%), and routine prenatal care (75.6%), revealing a significant lower rate of informed choice in routine prenatal care group [20]. The UK authors argued that this could be explained by less available counseling time in a routine setting, no requirement for a written consent and not discussing NIPT at multiple points [20]. This highlights the importance of counseling in facilitating informed decision making. The use of decision aids [31] and value clarification exercises [32] have also been shown to positively affect informed choice.

Our study found that women with intermediate and high levels of education were more likely to make an informed choice and women with religious affiliation were less likely to make an informed choice. For the latter group, the choice to decline fetal aneuploidy screening may simply have been in accordance with their religious beliefs, with women having lower levels of knowledge on screening, likely due to a lack of interest. Previous studies have shown that religious faith is an important factor in the decision to accept or decline fetal aneuploidy screening [33, 34]. The concept of informed choice has been the subject of criticism mainly because there is no uniform or validated approach to its determination. Scales and cut-offs that are used vary greatly between studies [18, 19, 28]. Results are therefore difficult to compare and should be interpreted with caution. As such, there is a need for innovation of the measure of informed choice.

### Perceived freedom to choose

The majority of the respondents did not feel pressure from society or others (partner, family, or provider) to accept or decline prenatal screening, although 15% of test-decliners perceived societal pressure to accept screening. In a Dutch qualitative study conducted before the introduction of first-tier NIPT in 2017, some women reported feeling pressured to accept or decline screening by media and society [35]. In contrast, a comparable study during the introduction of NIPT reported no noticeable pressure to accept or decline screening [36]. Similar to our results, a Canadian survey study found that the majority of pregnant women anticipated no personal (64%) or societal (62%) pressure to accept NIPT. In this study 24% of Canadian women anticipated feeling (some) societal pressure [37]. Both the social context and the framing of the offer of screening have been shown to influence women’s decision-making and uptake of screening. The Dutch prenatal screening offer is comprised of several unique elements aimed at promoting women’s informed decision-making and freedom to choose. First, there is a centralized national screening program which offers all women counseling for prenatal screening by a certified obstetric counselor. In the program there is great emphasis on “the right not to know” (i.e., pregnant women are first asked if they wish to receive information about prenatal screening for congenital conditions, and only women who say “yes” receive counseling), which has also been reported as an important factor influencing the relatively low rate of screening in the Netherlands [22]. In Denmark, where the offer of screening is framed positively and as an “opt-out” choice, uptake is much higher [22]. Other aspects explaining the relative low uptake in the Netherlands are the negative attitudes toward pregnancy termination, the postitive attitudes toward Down syndrome and costs of screening. In our study, for 16% of test-acceptors, one of the reasons to choose NIPT over FCT was because it is easy to do. Other studies have reported similar findings [14]. For women to experience freedom to choose, declining prenatal screening should remain an equal option. The majority of Dutch women still decline fetal aneuploidy screening, indicating that women experience the freedom to refrain from testing [38]. However, low uptake might also indicate a barrier to access, for example due to the out-of-pocket costs of screening (€175 for NIPT) as the costs of NIPT are not (fully) covered by the healthcare system in the Netherlands. A survey study among European healthcare providers indicated that the costs and lack of reimbursement policy were considered to be the primary barrier in NIPT uptake [9]. Similar concerns were reported by healthcare providers from Lebanon and Quebec [39].

### Personal and societal perspectives on Down syndrome

In our study, test-acceptors believed more often than test-decliners that children with Down syndrome are less accepted in society than other children. Moreover, test-acceptors less often thought that care and support for children with Down syndrome are well arranged in the Netherlands. Indirectly, these perceptions may cause some women to feel pressured to accept prenatal screening, impeding on their freedom to choose. In Canada, it was shown that over half of pregnant women were at least somewhat concerned that the routinization of NIPT might lead to a reduction in available resources for and have a negative impact on people with Down syndrome and their families [37]. Additionally, 70% of test-acceptors perceived it a great burden to raise a child with Down syndrome compared to 28.6% of test-decliners. Different personal perceptions of Down syndrome could be the result of women making a choice that is in line with their values and perspectives. It has, however, been argued that future parents should be provided with more balanced information regarding living with Down syndrome in order to make an informed decision [40]. Previous research among the Dutch general public indicated that a small sub-group thinks negatively toward declining NIPT and giving birth to a child with a disability [41]. This may cause some pregnant women to experience societal pressure to test. In our study, women were less likely to intend to terminate for Down syndrome than for Edwards or Patau syndrome, as was also shown in previous research [19, 41]. A possible explanation for this finding may be that the severity of conditions and potential burden of the conditions for both parents and the child, impacts the intention to terminate. Edwards and Patau syndrome are considered to have a higher severity and burden compared to Down syndrome [41]. A proportion of women use NIPT for informational purposes to prepare for a child with a disability [42].

### Strengths and limitations

Strengths of this study include a nationwide study sample. Of our respondents, 78% said they intended to accept prenatal screening either with NIPT or FCT. This does not concur with the actual uptake of screening in the Netherlands (46% in 2018) [38]. It has been shown that women of non-Dutch descent and women with low education levels are less likely to participate in fetal aneuploidy screening [43, 44]. The majority of our respondents were highly educated and of Dutch descent, which may have led to an overestimation of women who participated in screening and of the proportion of informed choice. The large variation in response rates between participating sites (2–89%), may have increased the underrepresentation of low-educated, non-Dutch women. Practices with the lowest response rates were often practices with a larger population of low-educated and non-Dutch pregnant women. More research is needed among non-Dutch women and women with lower education levels. Furthermore, the questionnaire was only available in Dutch, which may have resulted in selective withdrawal. While our survey measured intention to screen, actual uptake could not be confirmed. Some respondents may have changed their decision after filling in the questionnaire.

## Conclusion

Our study suggests high levels of informed decision-making and perceived freedom to choose regarding fetal aneuploidy screening with NIPT. The results revealed that there is little reason for concerns regarding routinization of prenatal screening after the implementation of first-tier NIPT in the Netherlands, based on pregnant women’s perspectives. Though our findings are specific to the Dutch prenatal screening context, they highlight the importance of responsible implementation of first-tier NIPT within a national prenatal screening program. Informed decision-making should be safeguarded with high-quality counseling, emphasizing personal values and freedom to choose, and ensuring that women make a value-consistent choice for fetal aneuploidy screening. In order for pregnant women to make an informed decision free from pressure, high-quality care and support for people with disabilities is crucial.

## Supplementary information

Appendix NIPT Consortium

Supplementary Files

## Data Availability

The datasets generated during and/or analyzed during the current study are not publicly available due to the fact that the preparation of a publication regarding the second questionnaire is currently still ongoing, but are available from the corresponding author on reasonable request.
