# Supplementary material for: Routinization of prenatal screening with the non-invasive prenatal test: pregnant women’s perspectives
Source: Eur J Hum Genet. 2021 Aug 13;30(6):661–8. doi: 10.1038/s41431-021-00940-8 (PMC9177612; doi:10.1038/s41431-021-00940-8)
Supplement: Supplementary file 1 — Appendix NIPT Consortium [file 41431_2021_940_MOESM1_ESM.pdf]

# Appendix: The Dutch NIPT Consortium.

*The Dutch NIPT Consortium consists of obstetric care givers, laboratory specialists and other professionals from:*

**Amsterdam UMC***
Clinical Genetics:
Dr. E.A. Sistermans (also for the Dutch Association of Clinical Genetic Diagnostic Laboratories (VKGL))
Prof. L. Henneman
Dr. A. Polstra
E. Voorhoeve MSc
S.L. Zelderen-Bhola MSc
Dr. E.M.J. Boon
Dr. M.P.R. Lombardi
I.M.C. Bakker MSc
E.J. Bradley BSc
C. Louwerens-Zintel BSc
M. Smit BSc
Dr. M.C. van Maarle
M.B. Tan-Sindhunata MSc
K. van der Meij MSc
Prof. H. Meij
Obstetrics and Gynaecology:
Dr. C.J. Bax (also for the Dutch Organization of Obstetrics and Gynaecology (NVOG))
Prof. E. Pajkrt
Dr. I.H. Linskens
Midwifery Science AVAG:
Dr. L. Martin
Dr. J.T. Gitsels-van der Wal

**Erasmus Medical Center, Rotterdam***
Clinical Genetics:
Dr. R.J.H. Galjaard (also for the Dutch Association of Clinical Geneticists (VKGN))
Dr. D. Van Opstal
Dr. M.I. Srebniak
Dr. F.M. Sarquis Jehee
I.H.I.M Hollink MSc
Dr. F. Sleutels
W. de Valk BSc
W.H. Deelen BSc
Dr. A.M.S. Joosten
Dr. K.E.M. Diderich
M.E. Redeker
Obstetrics and Gynaecology:
Dr. A.T.J.I Go
Dr. M.F.C.M. Knapen
Dr. S. Galjaard
Dr. A.K.E. Prinsen
Information & Technology:
A.P.G. Braat

**Leiden University Medical Center**
Clinical Genetics:
Dr. M.J.V. Hoffer
Dr. N.S. den Hollander
Obstetrics and Gynaecology:
Dr. E.J.T. Verweij
Dr. M.C. Haak

**Maastricht University Medical Center***
Clinical Genetics:
Dr. M.V.E. Macville
Dr. S.J.C. Stevens
Dr. A. van der Wijngaard
Dr. H. Scheffer
L.H. Houben
M.A.A. van Esch-Lennarts BSc
Prof. C.E.M. de Die-Smulders
Prof. H. Brunner
Obstetrics and Gynaecology:
M.J. Pieters MD (also for the Regional Centres Prenatal Screening in The Netherlands)
Dr. A.B.C. Coumans

**Radboud University Medical Center, Nijmegen**
Clinical Genetics:
Dr. D.F.C.M. Smeets
Dr. B.H.W. Faas
Dr. D.E.M. Oldeweghuis
T. Hofste BSc
I. Derks-Prinsen BSc
Dr. I. Feenstra
Obstetrics and Gynaecology:
Dr. E. Sikkel

**University Medical Center Groningen**
Clinical Genetics:
Dr. R.F. Suijkerbuijk
Dr. B. Sikkema-Raddatz
Prof. I.M. van Langen
Dr. K. Bouman
Obstetrics and Gynaecology:
L.K. Duin MD

**University Medical Center Utrecht**
Clinical Genetics:
Dr. G.H. Schuring-Blom
Dr. K.D. Lichtenbelt
Obstetrics and Gynaecology:
Prof. M.N. Bekker

**The Royal Dutch Organization of Midwives (KNOV)**
Dr. A.J.E.M. van der Ven

**VSOP Patient Alliance for Rare and Genetic Diseases**
E. van Vliet-Lachotzki MD

**Erfocentrum, Dutch National Genetic Resource and Information Centre**
J. Pot

**Dutch NIPT Consortium, project manager**
S. van 't Padje

***NIPT laboratories***

Date version: March 2021
