# Supplementary material for: Routinization of prenatal screening with the non-invasive prenatal test: pregnant women’s perspectives
Source: Eur J Hum Genet. 2021 Aug 13;30(6):661–8. doi: 10.1038/s41431-021-00940-8 (PMC9177612; doi:10.1038/s41431-021-00940-8)
Supplement: Supplementary file 2 — Supplementary Files [file 41431_2021_940_MOESM2_ESM.pdf]

Supplementary information

Table S1 Knowledge questions.

	**True %**	**False %**	**I don't know%**
All children with Down syndrome are mentally handicapped. *(true)*	**68.5**	18.4	12.1
The combined test consists of a nuchal translucency measurement of the fetus using ultrasound and a maternal blood test. *(true)*	**86.4**	8.0	5.7
An abnormal (unfavourable) NIPT result means that the child definitely has a trisomy 21,18 or 13. (*false*)	7.9	**86.0**	6.1
In the case of a normal (favourable) NIPT result, there is still a small chance that the child has a trisomy 21,18 or 13. (*true*)	**70.2**	19.0	10.8
The combined test can more accurately determine whether the child has Down syndrome than the NIPT. *(false)*	7.5	**86.4**	6.1
Amniocentesis/chorionic villus sampling will give certainty about the presence of Down syndrome in the child. (*true*)	**66.8**	17.7	15.5
Prenatal screening for Down syndrome is mandatory for everyone. *(false)*	0.4	**98.0**	1.6

Table S2 Measures of informed choice.

	Description	Items	Range	Mean (SD)	Reliability	Cut-off	Outcome
Knowledge	Knowledge regarding prenatal screening, NIPT, FCT, Down syndrome, invasive testing and the meaning of possible test results.	Seven true-false statements. ‘Do not know’ was treated as incorrect.	0-7	5.6 (1.2)	-	≥ 5	Good knowledge: 83.2%
Deliberation	Deliberation about the pros and cons of the alternatives	Six 5-point Likert scale items	6-30	22.9 (4.1)	0.822	>18	Deliberated: 87.7%
Attitude	Attitude towards prenatal screening for Down, Edwards and Patau syndrome	Five 5-point Likert scale items	5-25	19.4 (4.2)	0.826	5-11 = negative; 12-18 = neutral; 19-25 = positive	Negative: 6.2%; neutral: 26.8%; positive: 67.0%
Uptake	Intention to accept or decline prenatal screening	Choice of three options: no test, NIPT or FCT	-	-	-	-	NIPT: 80.3%; FCT: 2.1%; No test: 17.6%
Value consistency	Consistency between value (attitude) and behaviour (test-uptake)	calculated	-	-	-	-	Value-consistent: 99.2%
Informed choice	A choice made with good knowledge, deliberated and behaviourally consistent with attitude	calculated	-	-	-	-	Informed choice: 75.3%

FCT= First-trimester Combined Test; NIPT= Non-Invasive Prenatal Test; SD= Standard Deviation.

Table S3 Prediction model of informed choice.

Variable	B (SE)	OR (95%-CI)	P-value
**Crude model**			
**Maternal age**			
≤ 30*			
31-35	0.30 (0.25)	1.35 (0.83-2.21)	0.228
≥ 36	0.37 (0.32)	1.45 (0.77-2.73)	0.253
**Education level**			
Low*			
Intermediate	1.28 (0.57)	3.60 (1.19-10.89)	0.023
High	1.27 (0.55)	3.55 (1.21-10.38)	0.021
**Ethnicity**			
Dutch*			
Other-western	-0.29 (0.39)	0.75 (0.35-1.60)	0.459
Non-western	-0.55 (0.40)	0.58 (0.27-1.25)	0.162
**Religious affiliation**			
None*			
Religious	-0.52 (0.23)	0.60 (0.38-0.93)	0.023
**Health literacy**			
Adequate*			
Not adequate	0.11 (0.32)	1.11 (0.59-2.10)	0.744
**Parity**			
Nulliparous*			
Multiparous	-0.30 (0.23)	0.74 (0.47-1.17)	0.198
**Gestational age**			
≤ 10*			
11-14	0.26 (0.23)	1.30 (0.84-2.02)	0.246
≥ 15	-0.53 (0.56)	0.59 (0.20-1.75)	0.341
**Method of conception**			
Natural*			
Assisted	0.23 (0.42)	1.26 (0.55-2.90)	0.583
**Adjusted model**			
**Education level**			
Low*			
Intermediate	1.22 (0.54)	3.37 (1.16-9.77)	0.025
High	1.19 (0.52)	3.29 (1.19-9.12)	0.022
**Religious affiliation**			
None*			
Religious	-0.54 (0.22)	0.58 (0.38-0.90)	0.015

* Reference category; B = unstandardized Beta; CI = Confidence Interval; OR = Odds Ratio; SE = Standard Error. Hosmer and Lemeshow test: 0.997.

Table S4 Reasons for choosing NIPT (n=1652) or FCT (n=29).

**Reasons for choosing NIPT**	**Responses (% of cases)**	**Reasons for choosing FCT**	**Responses (% of cases)**
NIPT is more reliable than the FCT	480 (29.1%)	The possibility to detect other findings by ultrasound	9 (31.0%)
NIPT is a safe test without a miscarriage risk	308 (18.6%)	Because of the additional ultrasound	7 (24.1%)
NIPT is easy to do	265 (16.0%)	I am not eligible for NIPT	3 (10.3%)
NIPT can be done early in pregnancy	191 (11.6%)	It is a safe test without miscarriage risk	3 (10.3%)
The possibility to detect additional findings	167 (10.1%)	Because of the fast result	2 (6.9%)
The FCT gives a risk result	132 (8.0%)	My midwife or doctor recommends FCT	2 (6.9%)
The risk for follow-up testing is low	48 (2.9%)	Other	2 (6.9%)
My midwife or doctor recommends NIPT	28 (1.7%)	It is the standard test	1 (3.4%)
My partner, family or others prefer NIPT	25 (1.5%)	My partner, family or others prefer FCT	0 (0.0%)
Other	8 (0.5%)	I do not want to participate in scientific research for NIPT	0 (0.0%)

FCT= First-trimester Combined Test; NIPT= Non-Invasive Prenatal Test
